# Spatial distribution and ecological niche modeling of geographical spread of *Anopheles gambiae* complex in Nigeria using real time data

**DOI:** 10.1038/s41598-023-40929-5

**Published:** 2023-08-22

**Authors:** Adedapo Adeogun, Ayodele Samuel Babalola, Okefu Oyale Okoko, Tolulope Oyeniyi, Ahmed Omotayo, Romoke Tawakalitu Izekor, Oluwakemi Adetunji, Abiodun Olakiigbe, Olalekan Olagundoye, Monsuru Adeleke, Cynthia Ojianwuna, Dagona Adamu, Abdullahi Daskum, Jibrin Musa, Obadiah Sambo, Oduola Adedayo, Petrus Uchenna Inyama, Lazarus Samdi, Abiodun Obembe, Musa Dogara, Poloma Kennedy, Suleiman Mohammed, Rebecca Samuel, Chioma Amajoh, Musa Adesola, Mohammed Bala, Mary Esema, Mamudu Omo-Eboh, Marianne Sinka, Olufunmilayo Ajoke Idowu, Adeolu Ande, Israel Olayemi, Abdulsalami Yayo, Perpetua Uhomoibhi, Samson Awolola, Babatunde Salako

**Affiliations:** 1 Nigerian Institute of Medical Research, Yaba, Lagos, Nigeria; 2 Department of Biological Sciences, Lead City University, Ibadan, Oyo State Nigeria; 3 Department of Pure and Applied Zoology, Federal University of Agriculture, Abeokuta, Nigeria; 4 National Malaria Elimination Program, Federal Ministry of Health, Abuja, Nigeria; 5 Department of Zoology, Faculty of Basic and Applied Sciences, Osun State University, Osogbo, Nigeria; 6 Department of Animal and Environmental Biology, Delta State University, Delta, Nigeria; 7 Biology Research Laboratory, Federal University, Gashua/Yobe State University, Yobe State, Gashua, Nigeria; 8 Department of Biological Sciences, Taraba State University, Jalingo, Nigeria; 9 USAID/Vectorlink Project Nigeria, Abuja, Nigeria; 10 Department of Zoology, Kwara State University, Melete, Kwara Nigeria; 11 Department of Biological Sciences, Faculty of Science, Federal University, Jigawa State, Dutse, Nigeria; 12 Department of Zoology, Faculty of Science, Gombe State University, Gombe, Nigeria; 13 Department of Biology, Umaru Musa Yar’adua University, Batagarawa, Katsina State Nigeria; 14 Department of Zoology, Madibbo Adama University of Technology, Yola, Adamawa State Nigeria; 15 Community Vision, Abuja, Nigeria; 16 Department of Zoology, University of Oxford, Oxford, UK; 17 Department of Zoology, University of Ilorin, Ilorin, Kwara State Nigeria; 18 Department of Animal Biology, Federal University of Technology, Minna, Nigeria; 19 Centre for Infectious Disease Research, Bayero University, Kano, Nigeria

**Keywords:** Ecological modelling, Entomology

## Abstract

The need for evidence-based data, to inform policy decisions on malaria vector control interventions in Nigeria, necessitated the establishment of mosquito surveillance sites in a few States in Nigeria. In order to make evidence-based-decisions, predictive studies using available data becomes imperative. We therefore predict the distribution of the major members of the *Anopheles gambiae* s.l. in Nigeria. Immature stages of *Anopheles* were collected from 72 study locations which span throughout the year 2020 resulted in the identification of over 60,000 *Anopheline* mosquitoes. Of these, 716 breeding sites were identified with the presence of one or more vector species from the *An. gambiae* complex and were subsequently used for modelling the potential geographical distribution of these important malaria vectors. Maximum Entropy (MaxEnt) distribution modeling was used to predict their potentially suitable vector habitats across Nigeria. A total of 23 environmental variables (19 bioclimatic and four topographic) were used in the model resulting in maps of the potential geographical distribution of three dominant vector species under current climatic conditions. Members of the *An. gambiae* complex dominated the collections (98%) with *Anopheles stephensi, Anopheles coustani, Anopheles funestus, Anopheles moucheti, Anopheles nilli* also present. An almost equal distribution of the two efficient vectors of malaria, *An. gambiae* and *Anopheles coluzzii*, were observed across the 12 states included in the survey. *Anopheles gambiae* and *Anopheles coluzzii* had almost equal, well distributed habitat suitability patterns with the latter having a slight range expansion. However, the central part of Nigeria (Abuja) and some highly elevated areas (Jos) in the savannah appear not suitable for the proliferation of these species. The most suitable habitat for *Anopheles arabiensis* was mainly in the South-west and North-east. The results of this study provide a baseline allowing decision makers to monitor the distribution of these species and establish a management plan for future national mosquito surveillance and control programs in Nigeria.

## Introduction

*Anopheline* mosquitoes remain successful in their ability to adapt to their changing natural environments, expanding opportunities for the propagation of their species. The majority of these adaptive processes comes at a cost to both the mosquitoes and their hosts as they serve as vectors of various human pathogens including malaria parasites, filarial worms and arboviruses (arthropod-borne viruses). Of specific importance are the members of the *Anopheles gambiae* complex (*Anopheles coluzzii*, *Anopheles gambiae* and *Anopheles arabiensis*) which have evolved over time, adapting and responding to ecological factors^1,2^, to become the primary and efficient vectors of malaria parasites in Africa^3,4^. Much of the efforts at combating malaria has been through massive investments in vector control targeted at these species, highlighting the importance of understanding vector distribution and dynamics^5^.

While the members of the *An. gambiae* complex commonly occupy similar ecological niches^2^, *An. gambiae* is widespread throughout Africa and is known to be ancestral^6^ whereas *An. coluzzii* is found mainly in West and Central Africa^7^ and *An. arabiensis*, which shares similar ecological areas with *An. gambiae*, prefers drier environments^1^. Both *An. coluzzii* and *An. gambiae* are highly anthropophilic and anthropophagic^8,9^ but *An. arabiensis* is more zoophilic^10^. These differences in habitat and feeding preferences may result in ecological variables differentially driving the dispersal patterns of these malaria vectors which potentially lead to a complex system influencing malaria parasite spread^2^. Knowledge of how geographical factors influence the dispersal of malaria vectors may therefore help in understanding vector distribution and adaptation and provide the baseline for evidence-based control interventions.

The recent WHO report^5^ clearly points out the need to provide greater evidence and invest more in malaria vector control in-country. Although Nigeria has invested a lot in vector control through mass distributions of long-lasting insecticidal nets (LLINs), its estimated malaria prevalence has not improved significantly and it still reports up to 54% of the total number of malaria cases in Africa^5^. The recent malaria indicator survey^11^ clearly points out the need to provide more data on both environmental and genetic factors that contribute to the survival and adaptation of the vectors in the face of ongoing insecticide-based vector interventions. The thrust of National Malaria Elimination Program’s (NMEP- Department of Public Health at the Federal Ministry of Health, Abuja) strategies for malaria control has been the scaling up of universal access to parasitological confirmation of malaria, treatment at all service delivery levels and provision of high impact vector control interventions^12^. This therefore led to a paradigm shift in the coordination of malaria control activities in Nigeria. To this end, the NMEP has been coordinating translational and operational research activities on vector dynamics, ecology and vectoral capacities of *Anopheles* mosquitoes vector species in different parts of Nigeria. The findings of such research activities are expected to inform decisions on appropriate strategies for sustainable vector control in the country.

Malaria vector control across Africa is becoming more problematic due to the development of resistance to the pyrethroid insecticides which are the only class of insecticides approved for use on bed nets for malaria control, in West and East Africa. In Nigeria, documented cases of resistance to pyrethroids and other commonly used insecticides have persisted^13,14^, severely limiting targeted malaria prevention and control efforts. Despite all evidence showing that insecticide resistance amongst malaria vector species is a major setback towards malaria control at the global level, the available information within Nigeria is not enough to inform an evidenced-based decision towards national malaria control. Therefore, advances in vector control that identify and target the most productive larval habitat and breeding sites are essential^15^. Owing to the limited movement of mosquito immature stages compared with that of free-flying adult mosquitoes, control of the immature stages can be more efficient^16^. In order to be able to design and implement control measures directed at the larval stages such as larval source reduction and larviciding, an understanding of the spatial and temporal distribution of malaria mosquito larvae in different malaria transmission settings is important^17,18^.

A lack of contemporary *Anopheles* distributional data limits the capacity for distributional modeling, hindering effective vector control which subsequently poses a serious challenge in combating malaria in Nigeria. Spatial modelling, such as Ecological Niche Modeling (ENM), can be used to bridge knowledge gaps in the distribution of organisms. When applied to mosquito vectors, it identifies areas of suitable habitat for each species where data is lacking which allows evidence-based estimates to be made on the risk of malaria transmission in areas not covered by the current interventions^19,20^. An ecological niche can be described as the set of natural conditions (biotic and abiotic variables) in which a species is able to preserve reasonable population sizes without migration^21^. Ecological Niche Modeling is a useful tool that extracts and identifies the fundamental boundaries of a species’ niche at each occurrence location in a selected dataset from a suite of relevant environmental covariates. Predictions of Ecological Niche Modeling can infill the voids resulting from surveillance data shortages^22–27^. This study therefore presents the spatial distribution of malaria vectors in 12 states in Nigeria and developed an ecological niche model that address information gaps and provide evidence-based predictions for public health decision makers to guide future national surveillance and control programs.

## Materials and methods

### Study area

**Figure 1:**
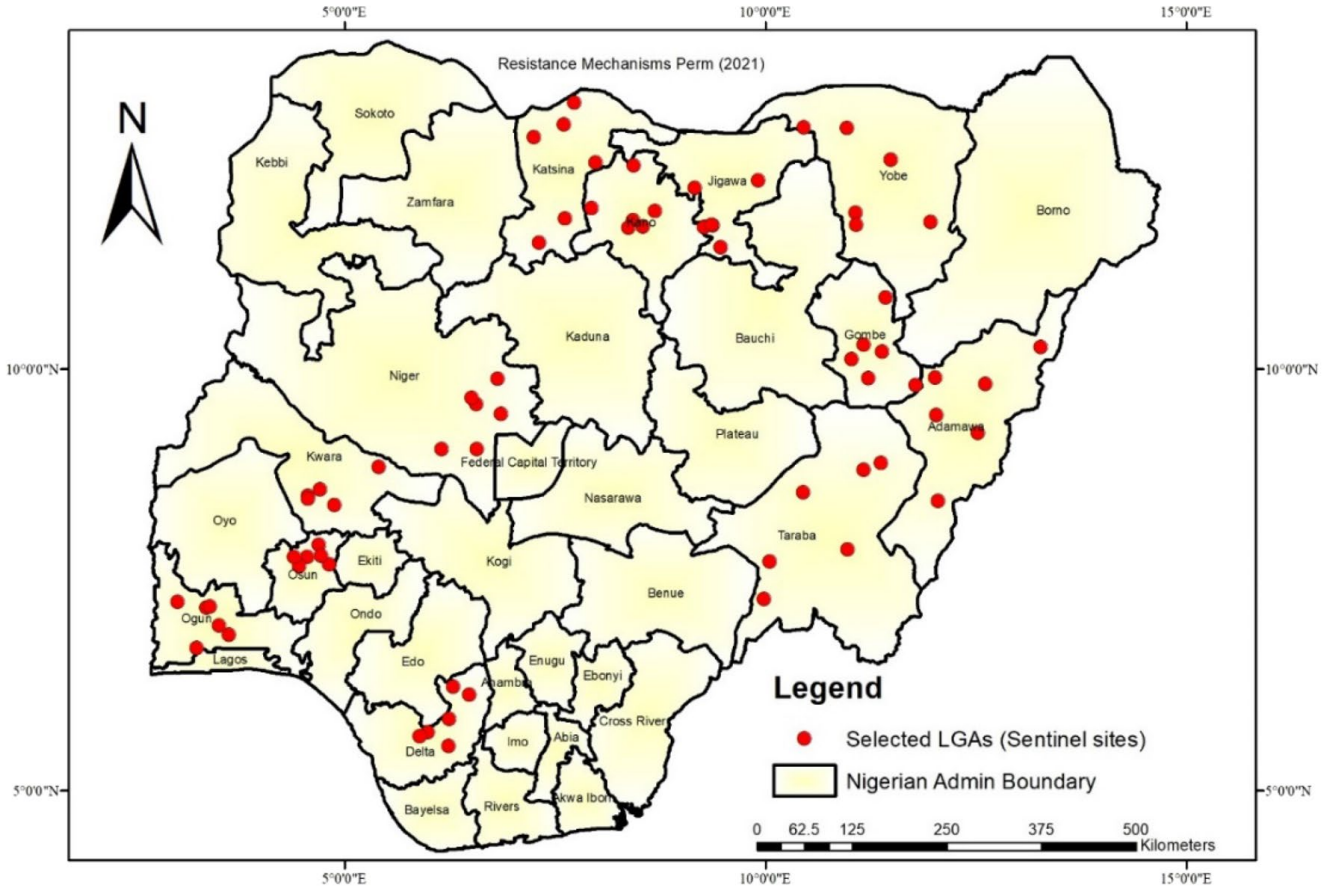
Map of Nigeria showing the sentinel sites from 12 selected states for the surveillance project. This figure was created by the authors in R programming software (R version 4.1.2, Vienna, Austria). Available at https://www.R-project.org/. The Nigerian shapefile was obtained from World BankDataCatalog () an Open license standardized resource of boundaries (i.e., state, county) for every country in the world.

Nigeria (9.0820° N, 8.6753° E) has 36 states plus FCT with an estimated population of 215,266,577^28^. Routine mosquito surveillance (Larval collection) was conducted in twelve states (Table 1) which span all the ecological zones in the country (Fig. 1). Furthermore, mosquitoes were collected from 6 local government areas (LGAs) from each state (making a total of 72 LGAs). Generally, the climate in the twelve States is typically tropical with two distinct seasons, rainy (May to October) and dry (November to April). The mean annual weather conditions in the three states range from 24.0 °C to 30.20 °C (Temperature), 31.1–85% (Relative Humidity) and 314 mm–1871 mm (Rainfall). In terms of vegetation, the states in the north consist of Guinea, Sudan and Sahel Savannah while the southern part includes the mangrove and the forest in addition to the Guinea Savannah (Table 1). The main method of vector control in the study areas is insecticide-treated bed nets (ITNs). In the past 5 years, more ITNs have been deployed in the states through free mass distributions, resulting into high coverage (over 70%) in the study areas.

**Table 1:** States, LGAs and Ecological zones of sites where mosquitoes were collected.

S/N	States	Local government areas	Ecozone
1	Adamawa	Yola North, Numan, Shelleng, Mubi North, Song, Ganye	Guinea & Sudan Savannah
2	Delta	Ika North East, Ukwuani, Aniocha South, Ethiope East, Okpe, Isoko North	Rainforest & Mangroove
3	Gombe	Gombe, Balanga, Billiri, Funakaye, Akko, Yamaltu-Deba	Sudan & Sahel savannah
4	Jigawa	Dutse, Birnin-Kudu, Ringim, Taura, Kafin Hausa, Auyo	Sudan& Sahel Savannah
5	Katsina	Malumfashi, Funtua, Kusada, Bindawa, Kaita, Batagarawa	Sudan & Guinea Savannah
6	Kano	Kura, Warawa, Garun-Mallam, Gwarzo, Bunkure, Makoda	Sudan Savannah
7	Kwara	Moro, Asa, Ilorin South, Ilorin East, Ilorin West, Ifelodun	Guinea Savanah
8	Niger	Katchia, Chachanga, Lapai, Bosso, Shiroro, Paikoro	Guinea
9	Taraba	Jalingo, Ardokola, Gassol, Bali, Donga, Takum	Guinea & Sudan Savanah
10	Ogun	Odeda, Obafemi Owode, Shagamu, Ado Odo Ota, Yewa North, Abeokuta South	Rainforest
11	Osun	Obokun, Boripe, Ede, Oriade, Oshogbo, Egbedore	Rainforest
12	Yobe	Damaturu, Potiskum, Nguru, Nnengere, Bursalli, Bade	Sudan & Sahel savannah

### Mosquito collection and identification

The World Health Organization (WHO) and Center for Disease Control (CDC)’s entomological protocols for field and laboratory studies on malaria vectors were employed in carrying out the studies. Mosquitoes were uniformly collected on weekly basis during the raining season (between may and October) in the year 2020 across all the states. Surveys of larval breeding sites were carried out in the selected LGAs across the 12 States (Table 1). *An.* larvae were collected from identified breeding sites such as permanent water bodies, rice fields, small water pools, or impoundments, using a dipping method described by WHO^29^ and the location of each breeding site was georeferenced using a GPS device. Collected larvae were transported to the insectaries (for each state) for rearing where they were reared to adulthood under ambient laboratory conditions and the emerged adults were identified morphologically by two entomological technicians and finally validated by the Principal Investigator (for each state) using the keys of Gilles and Coetzee^30^ under × 1000 Dino-lite HD color CMOS sensor high speed digital microscope (*model* number, AD4113T-12 V).

Mosquitoes belonging to the *An. gambiae* complex were further identified using the *An. gambiae* species-specific PCR^31^. DNA from specimens identified as *An. gambiae* were subjected to PCR assays for identification of *An. coluzzii* and *An. gambiae*^32^.

### Environmental data

Climatic variables such as temperature and precipitation influence species distributions at global and meso scales, topographic variables such as altitude and aspect have more influence at meso and topo-scales whereas land-cover variables such as percent canopy cover can influence distributions at the micro-scale^33,34^. Hence, climatic and topographic level variables were used here to predict the distribution of *An.* spp. in Nigeria.

**Figure 2:**
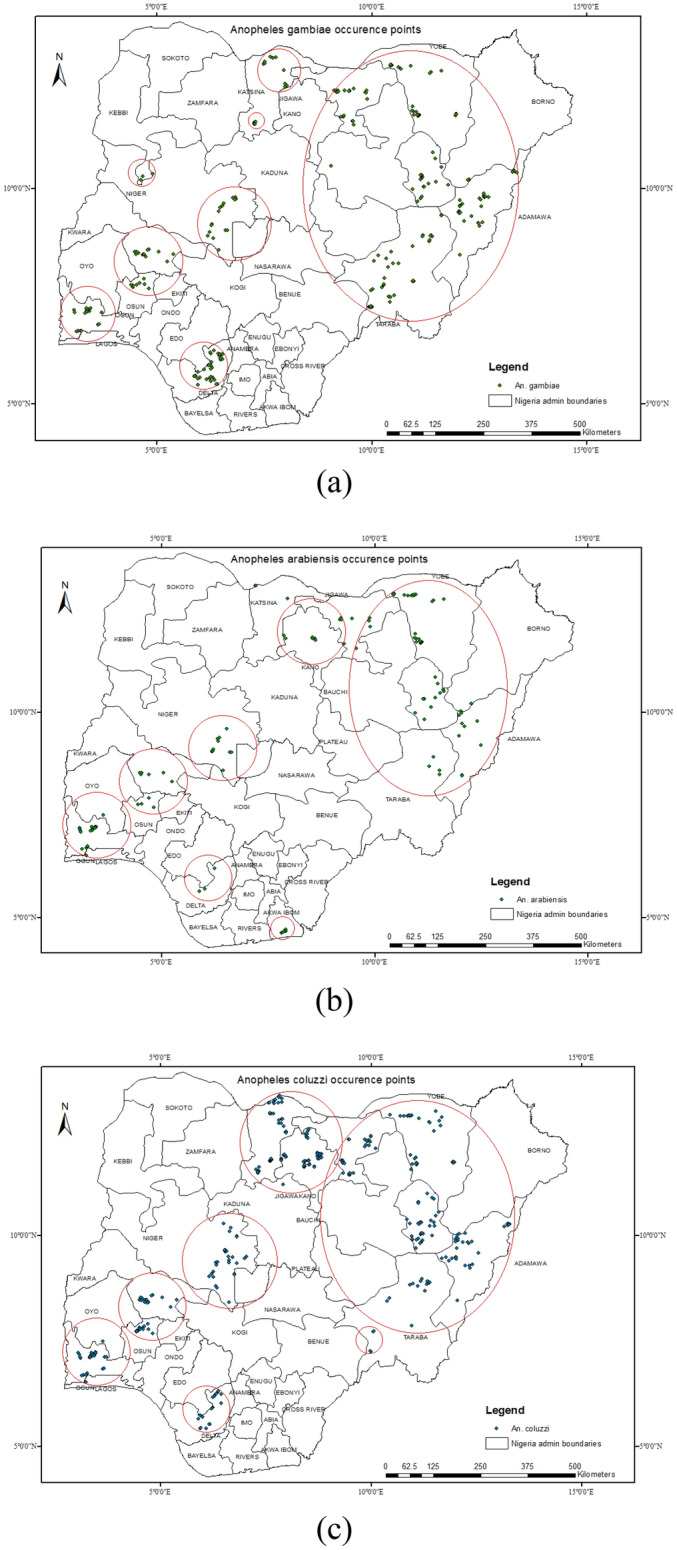
Map showing the positive occurrence records for each of the members of *An. gambiae* complex. (**a**) *An. gambiae* (**b**) *An. arabiensis* (**c**) *An. coluzzii.* This figure was created by the authors in R programming software (R version 4.1.2, Vienna, Austria). Available at https://www.R-project.org/. The Nigerian shapefile was obtained from World BankDataCatalog () an Open license standardized resource of boundaries (i.e., state, county) for every country in the world.

A total of 716 positive breeding sites were discovered throughout the 12 states for the members of *An. gambiae s.l.* To determine the possible distribution of *Anopheles* spp. in Nigeria, a total of 421, 132 and 279 positive occurrences for *An. coluzzii, An. arabiensis* and *An. gambiae* respectively from the field surveys were used in our ENM (Fig. 2). The autocorrelation problems were addressed by eliminating redundant presences on the scale of the bioclimatic variables used in each 1 × 1 km grid^35^. In addition, records for spatial autocorrelation were screened in ArcGIS 10.7.1 using average nearest neighbor analyses to remove spatially correlated data points^36,37^. After this selection, a total of 329, 86 and 212 positive occurrence points for *An. coluzzii, An. arabiensis* and *An. gambiae* respectively were used in our prediction model. We considered 19 environmental and four topographical variables as potential predictors of the target species habitat distribution^38,39^. These variables were chosen based on their biological relevance to the target species distributions^34,40–42^. The nineteen bioclimatic variables with a 2.5 min spatial resolution (about. 1 km^2^) were downloaded from the WorldClim database (http://www.worldclim.org/)^43^. Elevation data 1 km^2^-resolution was obtained from the Shuttle Radar Topography Mission (SRTM). The elevation data was used to generate slope, aspect, and hillshade (all in degrees) using the Spatial Analyst tool/surface in using ArcGIS 10.4.1 software.

The coordinates for all occurrence data were taken in decimal degrees (to four decimal places) and plotted using Google Earth to check for annotation errors. After downloading the climatic files, the Nigeria layer was extracted by using a boundary mask. After that, extracted files were converted to ASCII format via using ArcGIS 10.7.1 software to be used later with Maxent software.

All combinations of the 23 environmental and topographic variables have been tested for multi-collinearity through the calculation of R-squared in linear regression analysis in R software ver. 4.1.2. In this study, because some of these bioclimatic variables were strongly correlated (R^2^ ≥ 0.7), only those variables that showed little correlation with other predictors were retained; following^29,30^. A total of 12 environmental and topographical variables were selected in this study (R^2^ < 0.7) (Table 2).

**Table 2:** Environmental variables used for modeling the potential distribution of *Anopheles* spp. in the present study.

No	Variable	Code/Unit	Source
1	Annual mean temperature	Bio1 (°C)	WorldClim
2	Mean Diurnal Range (Mean of monthly (max temp—min temp))	Bio2 (°C)	WorldClim
3	Isothermality	Bio3 (°C)	WorldClim
4	Mean Temperature of Driest Quarter	Bio9 (°C)	WorldClim
5	Precipitation of the Wettest Month	Bio13 (mm)	WorldClim
6	Precipitation of the Driest Month	Bio14 (mm)	WorldClim
7	Precipitation of the Coldest Quarter	Bio19 (mm)	WorldClim
8	Elevation	–	SRTM
9	Slope	–	SRTM
10	Aspect	–	SRTM
11	Hillshade	–	SRTM

### Modelling

The modeling technique maximum entropy distribution or Maxent were used in this study; which has been found to be highly ranked among several different modeling methods^27,38,44^, and may continue to be effective even with small sample sizes^39,41,45–47^. For the study area, it only requires species presence data (not absence) and environmental variable (continuous or categorical) layers. We used the freely available Maxent software, version 3.3.3, which generates an estimate of the probability of the presence of the species that varies from 0 “unsuitable” to 0.99 “best habitat suitability”. ASCII files of the 8 selected environmental variables and a CSV file of species presence coordinates in decimal degrees were used to create the module. Maxent’s performance was assessed using a threshold independent Receiver-Operating Characteristic (ROC) analysis and Area Under Receiver- Operating Characteristic Curve (AUC) values (0.5 = random to 1 = perfect discrimination). The algorithm either runs 1000 iterations of these processes or continues until convergence is reached (threshold 0.00001).

For the model, the relative importance of each environmental predictor was evaluated using the percentage contribution of the Jackknife test, which is the best index for small sample sizes^41^. The default logistic output format was chosen, i.e. related to the probability of suitable conditions, ranging from 0 to 1. A total of 75% of the location point data were used for training, and the remaining 25% to test the predictive ability of the model (Model validation), in addition 10 replicates were considered. Average and Standard deviation values for training and test AUC for the 10 models were extracted from the Maxent text result output. The ASCII output map for the average model for the target species was loaded in ArcGIS 10.7.1 where the prediction models of habitat suitability were divided based on Choudhury et al.^48^ into 5 classes; very low (0–0.1), low (> 0.1–0.2), moderate (> 0.2–0.4), high (> 0.4–0.6) and very high (> 0.6) using natural breaks in the symbology tools to produce the habitat suitability model picture^49^.

### Ethical approval

Ethical clearance for this study was obtained from the Ethics review committee, Federal Ministry of Health. All methods including mosquito larva collection and breeding, laboratory analysis and data management were performed in accordance with the 1964 Declarations of Helsinki.

## Results

### Spatial distribution of Anopheline species collected from the 12 states

**Figure 3:**
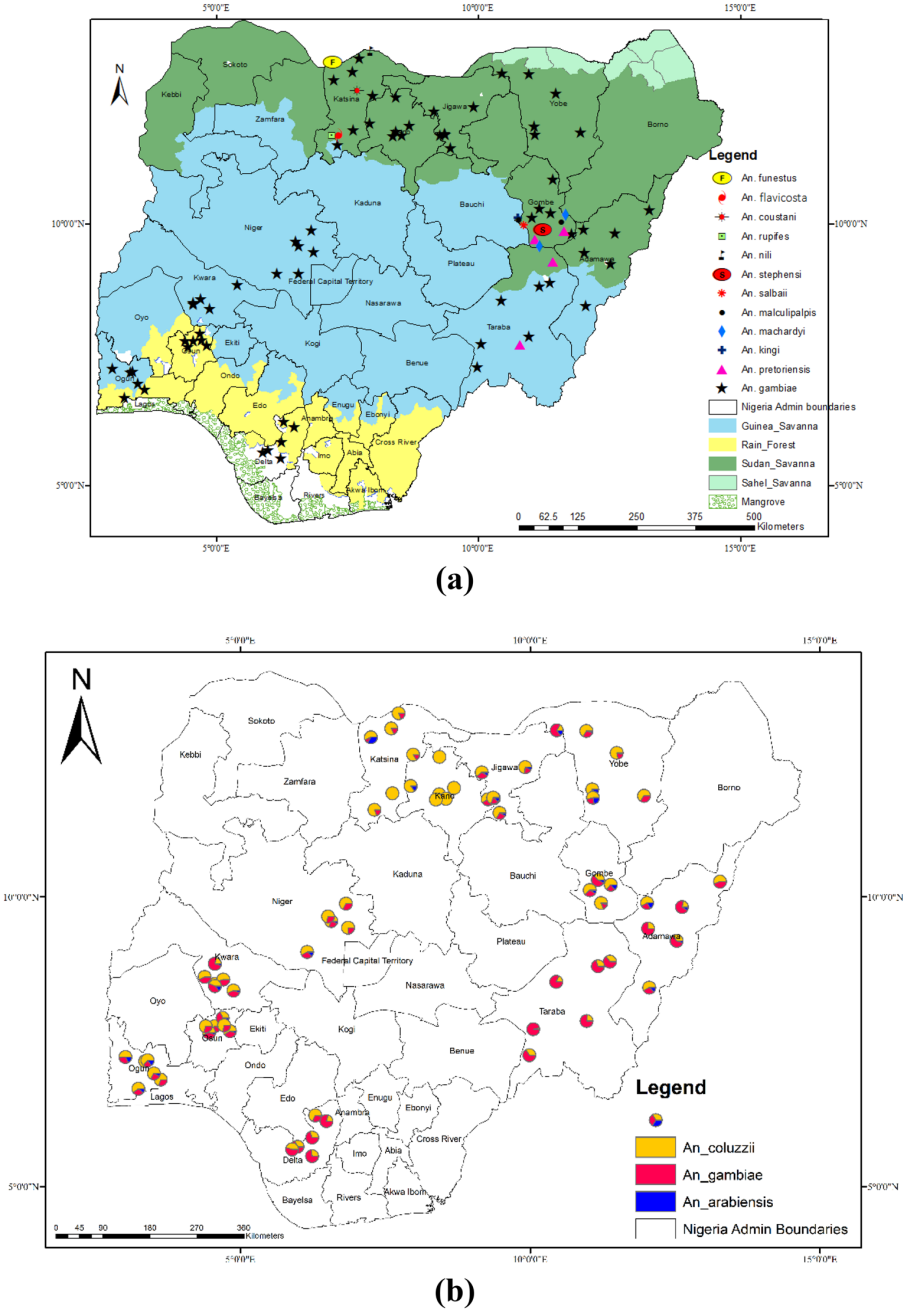
Spatial distribution of *An.* species collected from the 12 states. (**a**) Distribution of *Anopheline* mosquitoes in Nigeria. (**b**) Distribution of the members of the *An. gambiae s.l.* complex from the collection sites. This figure was created by the authors in R programming software (R version 4.1.2, Vienna, Austria). Available at https://www.R-project.org/. The Nigerian shapefile was obtained from World BankDataCatalog () an Open license standardized resource of boundaries (i.e., state, county) for every country in the world.

Members of the *An. gambiae* complex were the predominant species collected across all the LGAs from the 12 states, constituting up to 98% of total *Anopheles* collected. Furthermore, other *Anopheline*s (less than 2% in each state, see Supplementary File 1) notably, *An. salbaii, An. pretoriensis, An. funestus, An. flavicosta, An. coustani*, *An*. *machardyi* etc., were encountered in three northern states in addition the *An. gambiae* complex. It is noteworthy, to state that a total of 14(0.3%) *An. stephensi,* an invasive species and efficient vector for urban malaria transmission was also encountered in Gombe State (though only at 2 sites in one LGA)^50^. Members of the *An. gambiae* complex identified were *An. coluzzii*, *An. gambiae* and *An. arabiensis*. These species were variably distributed in the 12 states with *An. coluzzii* dominating the majority of the collections in all areas, followed by *An. gambiae* and then *An. arabiensis* (Fig. 3).

### Potential habitat distribution for the members of *An. gambiae* complex in Nigeria

**Figure 4:**
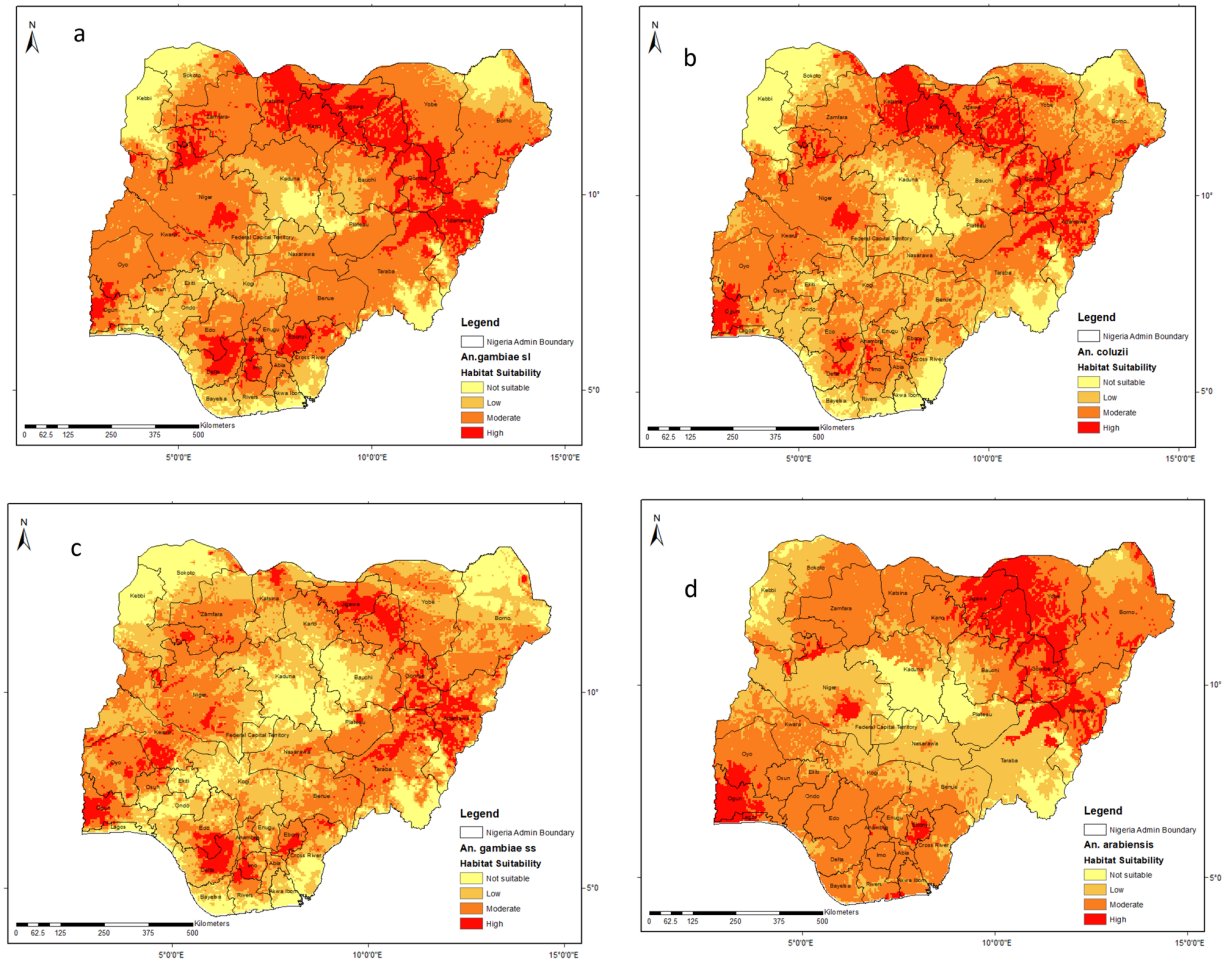
Predictive maps of geographical spread of the members of *An. gambiae s.l.* This figure was created by the authors in R programming software (R version 4.1.2, Vienna, Austria). Available at https://www.R-project.org/. The Nigerian shapefile was obtained from World BankDataCatalog () an Open license standardized resource of boundaries (i.e., state, county) for every country in the world.

The potential habitat distribution of the members of *An. gambiae* complex*.,* are presented in Fig. 4. The results showed that suitable larval habitats for *An. gambiae* complex are widely distributed across the country in varying proportions in (Fig. 4a). Habitat suitability for each of the members of the *An. gambiae* complex (*An. coluzzii, An. gambiae* and *An. arabiensis*) are highlighted below;

Our model predicts habitat suitability for *An. coluzzii* across eight states (Ogun, Lagos, Katsina, Kano, Jigawa, Bauchi, Gombe and Adamawa States). Conversely, it indicated only a few selected areas of Niger, Sokoto, Oyo, Yobe, Borno, Taraba, Kebbi, Delta, Anambra, Imo and Ebonyi States were highly suitable for *An. coluzzii*. Kebbi, Sokoto, Kaduna, Bauchi, Akwa-Ibom, Rivers and Bayelsa states were not predicted to be suitable for *An. coluzzii.* Oyo, Kwara, Osun, Niger, Zamfara, FCT, Nasarawa, Plateau, Yobe, Borno, Kogi, Edo, Imo, Ebonyi, Cross River, and Taraba States were moderately suitable for *An. coluzzii* (Fig. 4b).

For *An. gambiae,* larger part of Ogun, Delta, Kwara, Imo, Ebonyi, Adamawa and Gombe States were found to be highly suitable. Larger part of Oyo, Niger, Zamfara, Katsina, Kano, Yobe, Borno, Taraba, Benue, Nasarawa, and Plateau States were predicted to be moderately suitable for *An. gambiae,* while larger parts of Kebbi, Sokoto, Kaduna, Bauchi, Kogi, Ekiti, Ondo, Bayelsa, Akwa-Ibom, and FCT seems to be unsuitable for *An.* gambiae (Fig. 4c).

Larval habitats suitable for *An. arabiensis* were found in all parts of three states (Ogun, Lagos, Ebonyi) out of the 36 states. Also, larger parts of Jigawa, Yobe, Gombe, and Bauchi states were found to be highly suitable for *An. arabiensis*. Few parts of Oyo (especially in areas bordering Ogun), Niger, Taraba, and Borno states were highly suitable for *An. arabiensis.* Larger parts of Taraba, Plateau, Kaduna, Kebbi, FCT, Nasarawa, Niger, Sokoto and Benue states were predicted to be not suitable for *An. arabiensis*, while the remaining 20 states were moderately suitable for *An. arabiensis* (Fig. 4d).

### Model performance and influencing factors

The average percent contribution (PC) and permutation importance (PI) of the 11variables used in the modeling of the members of *An. gambiae s.l.* distribution in this study were also assessed and highlighted below;

### An. coluzzii

In this study, precipitation of the coldest quarter had the highest contribution with PC and PI of 25.2 and 29 respectively, followed by annual mean temperature PC of 22.1 and PI of 11 and mean temperature of driest quarter with PC of 13.7 and PI of 9.8. The results showed that these variables are strong predictors of *An. coluzzii* distribution in Nigeria, accounting for over 70% of the variations in distribution observed (Table 3).

**Table 3:** Average percent contribution and permutation importance of the variables used in the modeling of *An. coluzzii*, *An. gambiae* and *An. arabiensis* distribution.

Variable	Percent contribution		
	*An. coluzzii*	*An. gambiae*	*An. Arabiensis*
Precipitation of the Coldest Quarter	25.2^a^	10.1^f^	31.1^b^
Annual Mean Temperature	22.1^b^	16.6^a^	13^c^
Mean Temperature of Driest Quarter	13.7^c^	3.9^h^	36.3^a^
Isothermality (BIO/BIO7) (*100)	11.9^d^	14.6^c^	4.9^e^
Precipitation of the Wettest Month	8.4^e^	12.7^d^	0.3^h^
Mean Diurnal Range (Mean of monthly (max temp -min temp)	5^f^	16.1^b^	1.4^g^
Aspect	4.5^g^	3.1^i^	4^f^
Precipitation of the Driest Month	3.3^h^	11.3^e^	0.3^h^
Elevation	2.8^i^	8^g^	8.5^d^
Hillshade	2.3^j^	1.5^k^	0.1^k^
Slope	1^k^	2^j^	0.2^j^

Superscripts (alphabets) are assigned to each value in a column in descending order of percentage contributions, such that value with superscript a connotes the environmental variable with the highest contribution and value with superscript k is the environmental variable with the lowest contribution of each of the sibling species.

**Figure 5:**
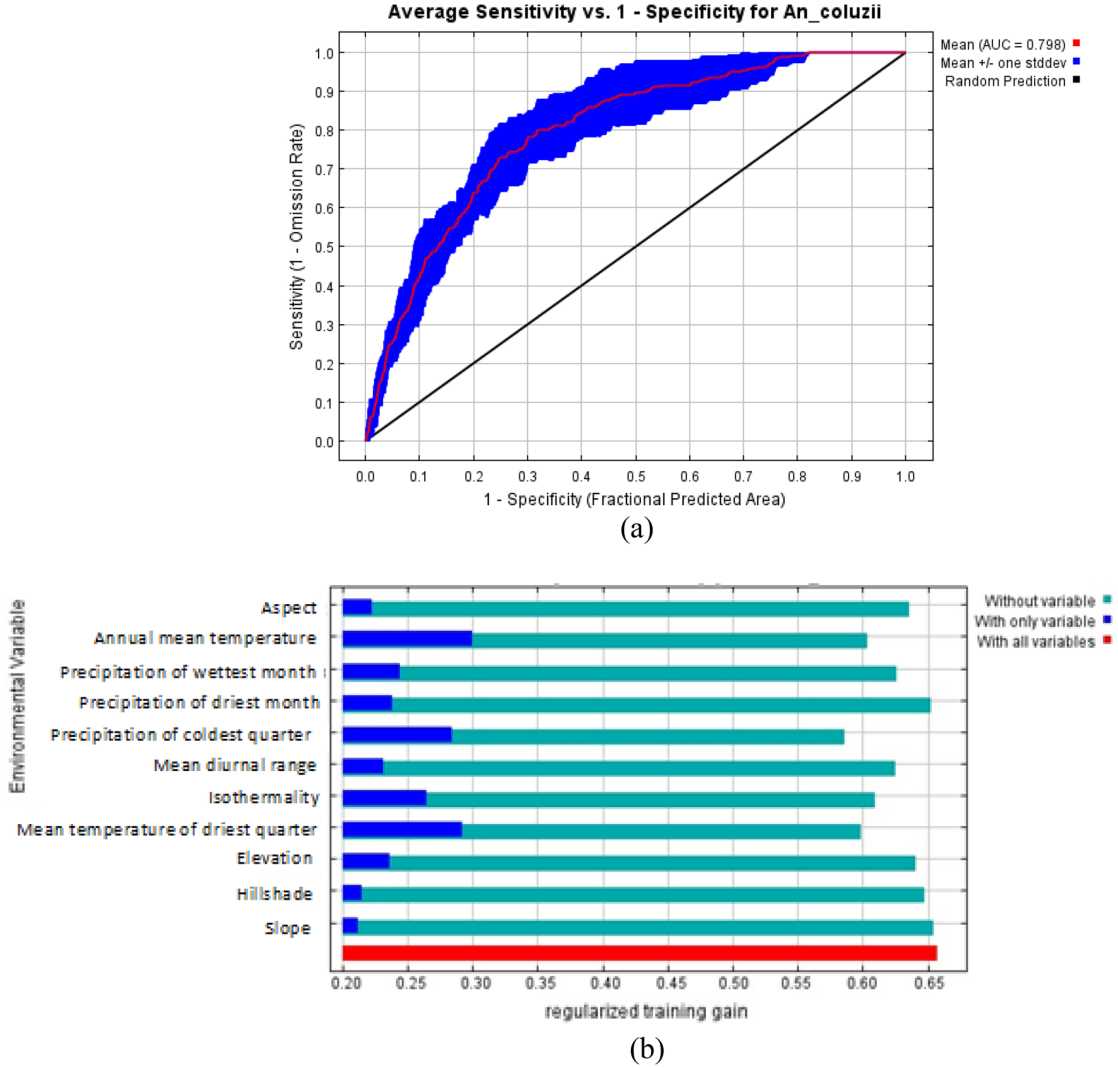
Estimation of model performance for *An. coluzzii* (**a**) Area under the curve (AUC) for *An. coluzzii* distribution. Red line indicates the mean value for 10 MaxEnt replicate runs. (**b**) Jackknife analysis for regularized training gain.

The ROC curve obtained as an average of the 10 replications runs is shown in Fig. 5a, and specificity was calculated. The average and standard deviation of the AUC for the 10 replicate runs was 0.798 ± 0.01. This value shows an excellent performance of the model as an AUC value of greater than 0.70 shows higher sensitivity and specificity for the presence of *An*. *coluzzii.*

The relative importance of each variable to the distribution of *An. coluzzii* was also computed with the Jackknife test in Fig. 5b which gave a training gain of 0.67. The Jackknife test showed that mean temperature of the driest quarter and annual mean temperature are the two variables that will increase the gain the most when used alone. The Jackknife test also showed that mean temperature of the driest quarter and precipitation of the coldest quarter will decrease the gain the most when removed from the model.

**Figure 6:**
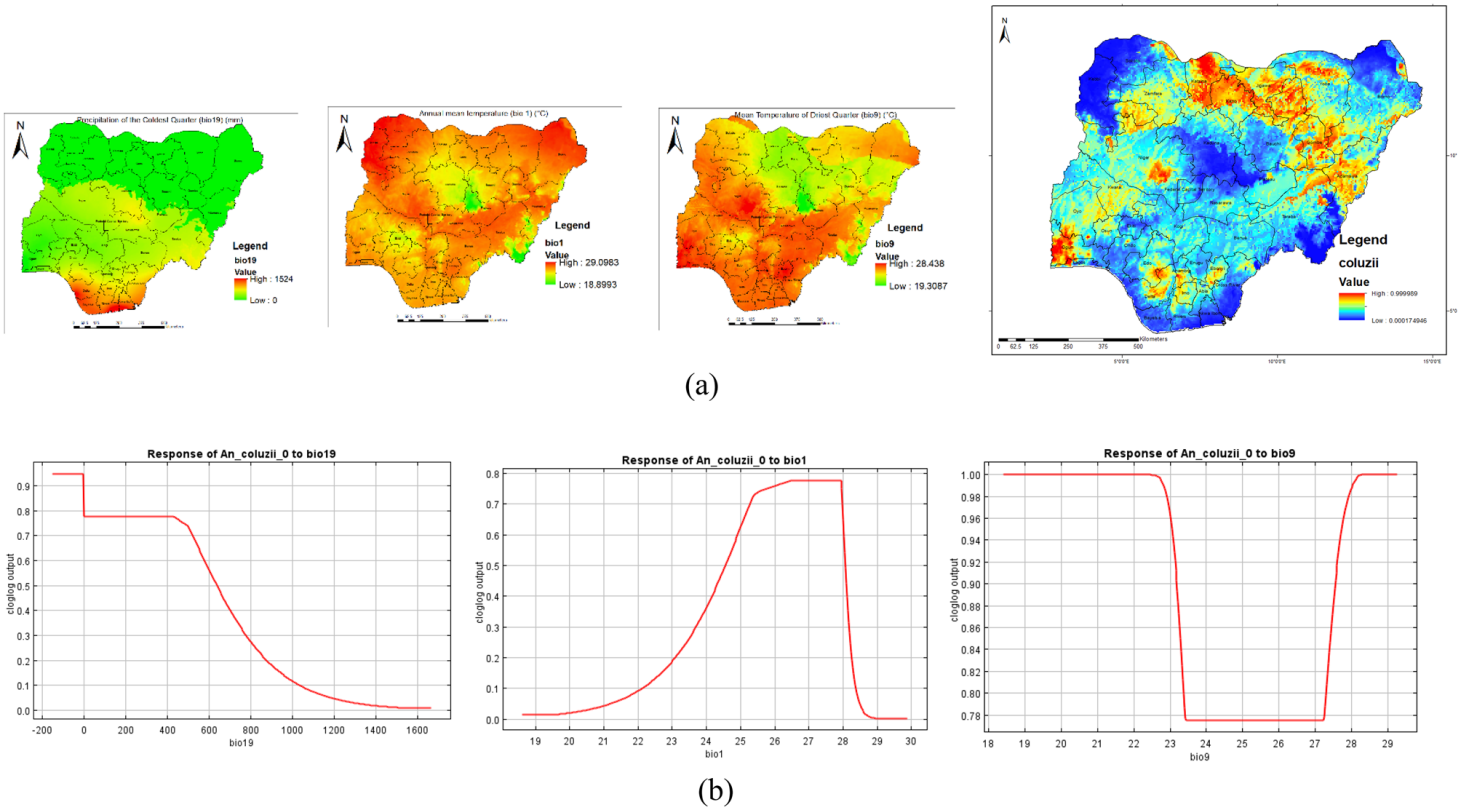
Estimates of the highest contributing variables that determines the geographical distribution of *An. coluzzii* (**a**) The highest environmental variables that estimate to control the geographical distribution of *An. coluzzii* in Nigeria. Variable contributions (precipitation of coldest quarter, annual mean temperature and mean temperature of driest quarter), (**b**) Response curves of three environmental predictors used in MaxEnt model for *An. coluzzii*. This figure was created by the authors in R programming software (R version 4.1.2, Vienna, Austria). Available at https://www.R-project.org/. The Nigerian shapefile was obtained from World BankDataCatalog () an Open license standardized resource of boundaries (i.e., state, county) for every country in the world.

Figure 6 shows the main highest estimated environmental variables (contributions) that determines the distribution of *An. coluzzii* in Nigeria. Spatial distribution analysis was done to determine the geographical variability with regards to the selected environmental variables in the country. The response curves of three variables to *An. coluzzii* habitat suitability are shown in Fig. 6a. We found out that mean temperature of the driest quarter ranged from 19.3 to 28.4 °C, precipitation of the coldest quarter ranged from 0 to 1524 mm while annual mean temperature ranged from 18.9 to 29.1 °C. The response curves showed that between mean temperature of the driest quarter of 19 and 22 °C favors the potential distribution of *An. coluzzii.* Similarly, precipitation of the coldest quarter of − 200 to 400 mm significantly and potentially favoured the distribution of *An. coluzzii* while annual mean temperature ranging between 26 and 28 °C significantly favoured the distribution of *An. coluzzii* in Nigeria (Fig. 6b).

### An. gambiae

In this study, annual mean temperature had the highest contribution with PC and PI of 16.6 and 7.2 respectively, followed by mean diurnal range PC of 16.1 and PI of 28.7 and isothermality with PC of 14.6 and PI of 25.2. The results showed that these variables are strong predictors of *An. gambiae* distribution in Nigeria, accounting for over 70% of the variations in distribution observed (Table 3).

**Figure 7:**
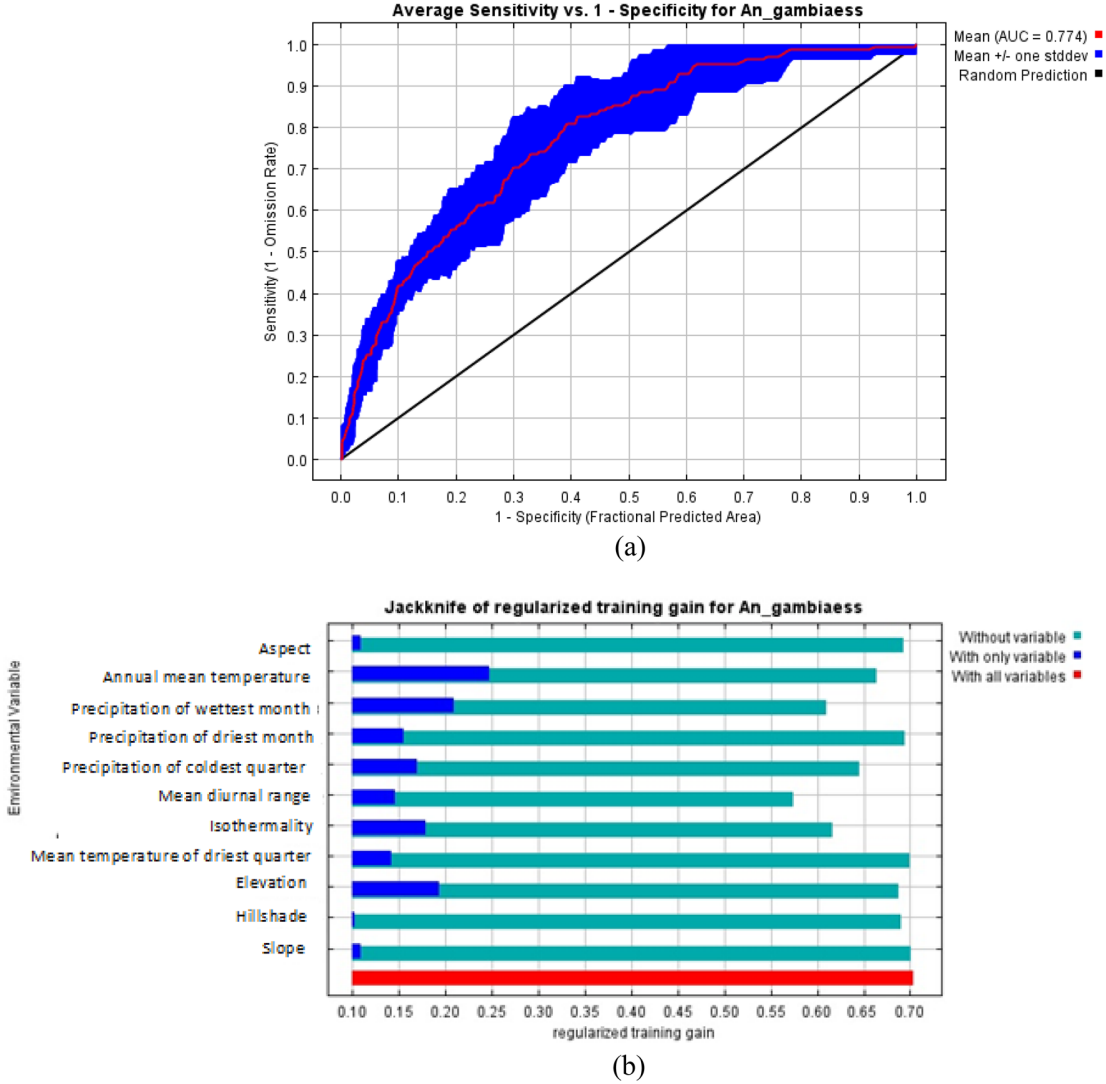
Estimation of model performance for *An. gambiae* (**a**) Area under the curve (AUC) for *An. gambiae* distribution. Red line indicates the mean value for 10 MaxEnt replicate runs. (**b**) Jacknife analysis for regularized training gain.

The ROC curve obtained as an average of the 10 replications runs is shown in Fig. 7a, and specificity was calculated. The average and standard deviation of the AUC for the 10 replicate runs was 0.774 ± 0.01. This value shows an excellent performance of the model as an AUC value of greater than 0.70 shows higher sensitivity and specificity for the presence of *An. gambiae.*

The relative importance of each variable to the distribution of *An. gambiae* was also computed with the Jackknife test in Fig. 7b which gave a training gain of 0.71. The Jackknife test showed that precipitation of the wettest month and annual mean temperature are the two variables that will increase the gain the most when used alone. The Jackknife test also showed that mean diurnal range and precipitation of the wettest month will decrease the gain the most when removed from the model.

**Figure 8:**
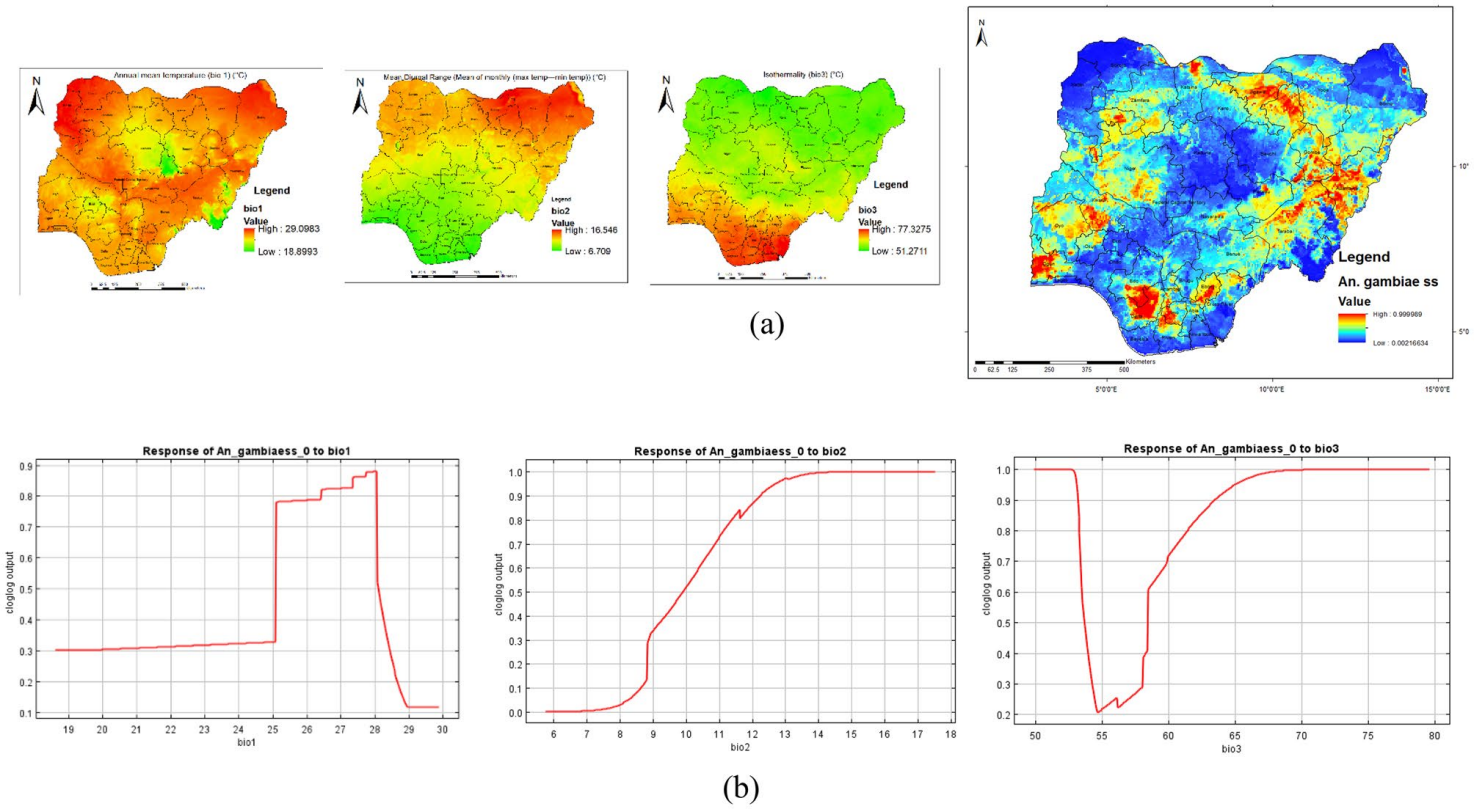
Estimates of the highest contributing variables that determines the geographical distribution of *An. gambiae* (**a**) The highest environmental variables that estimate to control the geographical distribution of *An. gambiae* in Nigeria. Variable contributions (annual mean temperature and mean diurnal range and isothermality), (**b**) Response curves of three environmental predictors used in MaxEnt model for *An. gambiae*. This figure was created by the authors in R programming software (R version 4.1.2, Vienna, Austria). Available at https://www.R-project.org/. The Nigerian shapefile was obtained from World BankDataCatalog () an Open license standardized resource of boundaries (i.e., state, county) for every country in the world.

Figure 8a shows the main highest estimated environmental variables (contributions) that determines the distribution of *An. gambiae* in Nigeria. Spatial distribution analysis was done to determine the geographical variability with regards to the selected environmental variables in the country. The response curves of three variables to *An. gambiae* habitat suitability are shown in Fig. 10a. We found out that annual mean temperature ranged from 18.9 to 29.1 °C, mean diurnal range ranged from 6.7 to 16.6 °C while isothermality ranged from 51.3 to 77.3 °C. The response curves showed that between annual mean temperature of 25 and 28 °C favors the potential distribution of *An. gambiae.* Similarly, mean diurnal range of 11 to 18 °C significantly and potentially favoured the distribution of *An. gambiae.* while isothermality between 65 and 80 °C significantly favoured the distribution of *An. gambiae* in Nigeria (Fig. 8b).

### An. arabiensis

In this study, mean temperature of the driest quarter had the highest contribution with PC and PI of 36.3 and 8.9 respectively, followed by precipitation of the coldest quarter with PC of 31.1 and PI of 30.5 and annual mean temperature with PC of 13 and PI of 16.8. The results showed that these variables are strong predictors of *An. arabiensis* distribution in Nigeria, accounting for over 80% of the variations in distribution observed (Table 3).

**Figure 9:**
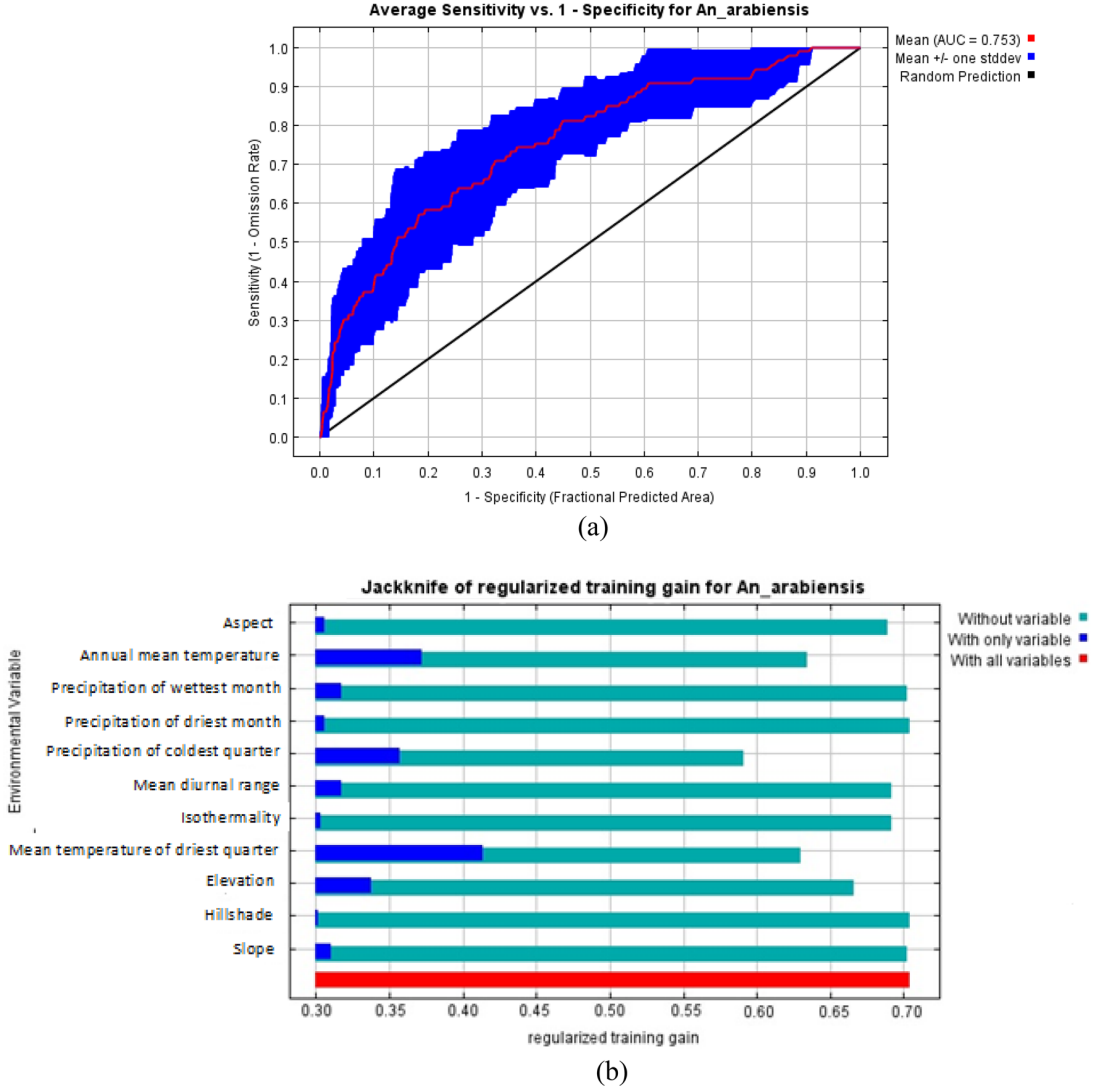
Estimation of model performance for *An. arabiensis* (**a**) Area under the curve (AUC) for *An. arabiensis* distribution. Red line indicates the mean value for 10 MaxEnt replicate runs. (**b**) Jackknife analysis for regularized training gain. The dark blue, light blue and red bars represent results of the model with each individual variable, all the remaining variables and all variables respectively.

The ROC curve obtained as an average of the 10 replications runs is shown in Fig. 9a, and specificity was calculated. The average and standard deviation of the AUC for the 10 replicate runs was 0.753 ± 0.01. This value shows an excellent performance of the model as an AUC value of greater than 0.70 shows higher sensitivity and specificity for the presence of *An*. *arabiensis.*

The relative importance of each variable to the distribution of *An. arabiensis* was also computed with the Jackknife test in Fig. 9b which gave a training gain of 0.72. The Jackknife test showed that mean temperature of the driest quarter and annual mean temperature are the two variables that will increase the gain the most when used alone. The Jackknife test also showed that precipitation of the coldest quarter will decrease the gain the most when removed from the model.

**Figure 10:**
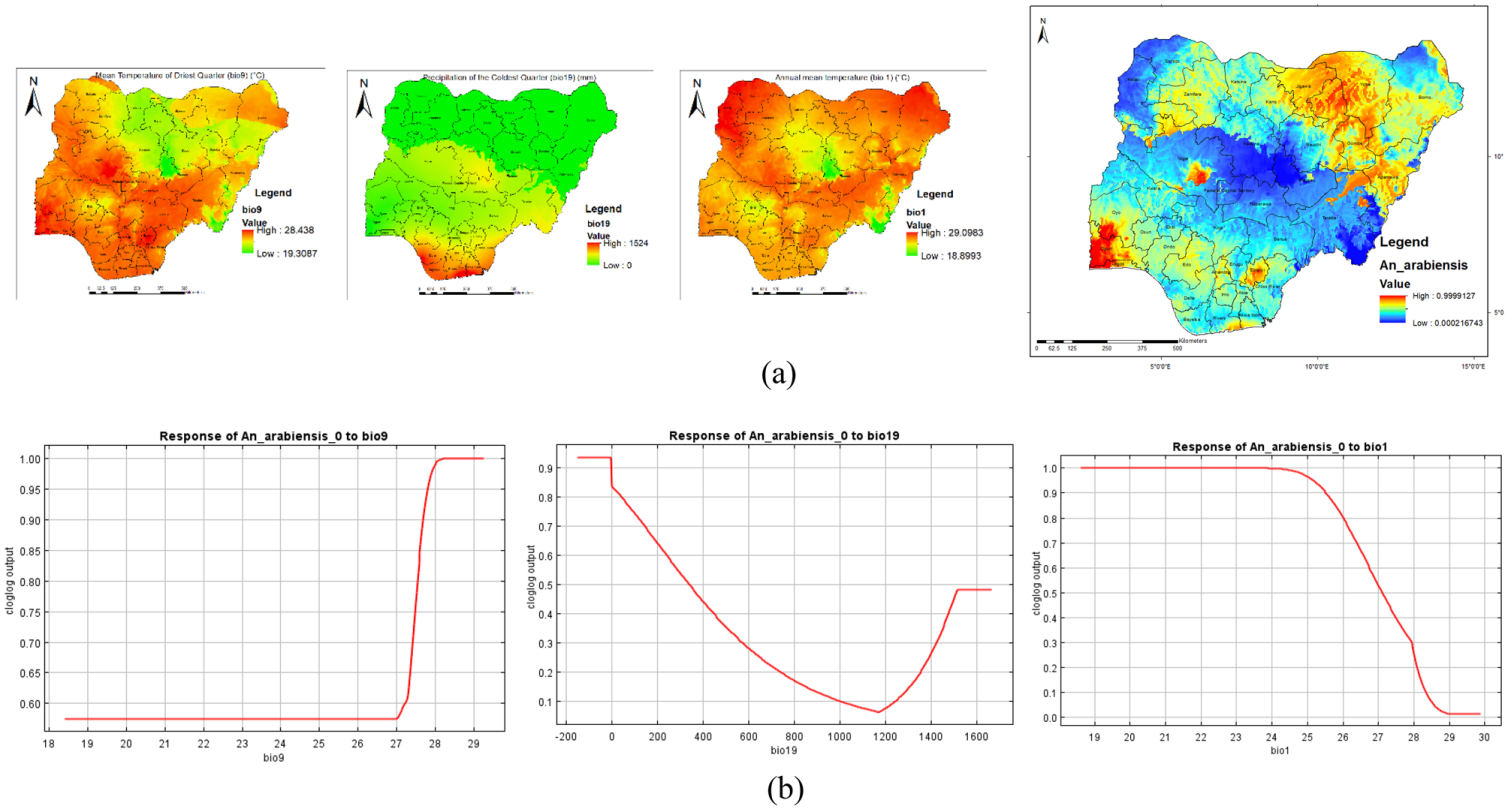
Estimates of the highest contributing variables that determines the geographical distribution of *An. arabiensis* (**a**) The highest environmental variables that estimate to control the geographical distribution of *An. arabiensis* in Nigeria. Variable contributions (mean temperature of driest quarter, precipitation of coldest quarter and annual mean temperature), (**b**) Response curves of three environmental predictors used in MaxEnt model for *An. arabiensis*. This figure was created by the authors in R programming software (R version 4.1.2, Vienna, Austria). Available at https://www.R-project.org/. The Nigerian shapefile was obtained from World BankDataCatalog () an Open license standardized resource of boundaries (i.e., state, county) for every country in the world.

Figure 10a shows the main highest estimated environmental variables (contributions) that determines the distribution of *An. arabiensis* in Nigeria. Spatial distribution analysis was done to determine the geographical variability with regards to the selected environmental variables in the country. The response curves of three variables to *An. arabiensis* habitat suitability are shown in Fig. 10b. We found out that mean temperature of the driest quarter ranged from 19.3 to 28.4° C, precipitation of the coldest quarter ranged from 0 to 1524 mm while annual mean temperature ranged from 18.9 to 29.1° C. The response curves showed that between mean temperature of the driest quarter of 28 and 29° C favors the potential distribution of *An. arabiensis.* Similarly, precipitation of the coldest quarter of 0 to 200 mm significantly and potentially favoured the distribution of *An. arabiensis* while annual mean temperature ranging between 19 and 25 °C significantly favoured the distribution of *An. arabiensis* in Nigeria (Fig. 10b).

### Relationship between the strongest environmental predictors of the distribution of the three *An.* species

**Figure 11:**
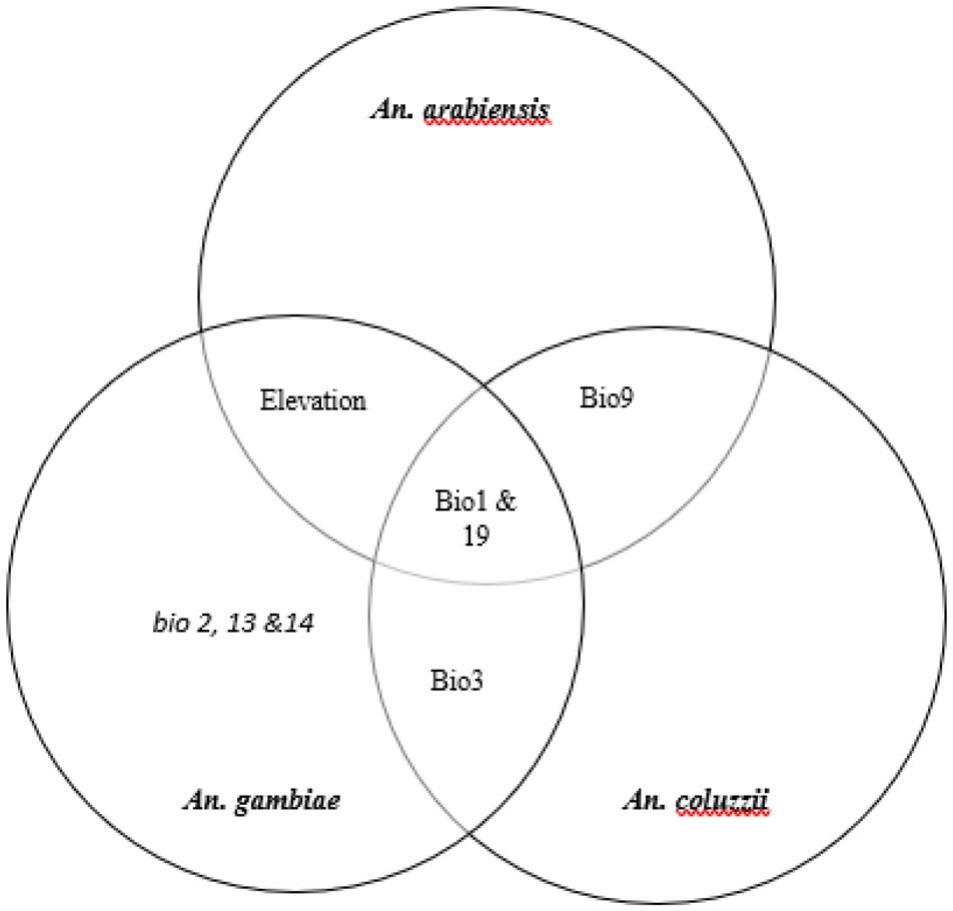
Relationship between the strongest environmental predictors of the distribution of the three *Anopheles* species.

Annual mean temperature and precipitation of the coldest quarter were common predictors for the three species, while isothermality is peculiar to both *An. gambiae and An. coluzzii.* Furthermore*,* mean temperature of driest quarter is common to both *An. coluzzii* and *An. arabiensis* while elevation is a common predictor to both *An. gambiae and An. arabiensis.* However, *An. gambiae* is the only species to have three environmental variables; mean diurnal range, precipitation of the wettest month and precipitation of the driest month as predictors peculiar to it alone (Fig. 11).

## Discussion

Vector surveillance is a very germane component of planning, implementation, monitoring and evaluation of vector control interventions. Having a holistic view of the distribution of major malaria vector populations for an entire country is a valuable asset for winning the battle against malaria transmission. Our work presents occurrence data for three malaria vectors in 12 states of Nigeria and a predicted distribution for the entire country using the maximum entropy algorithm approach.

Okorie et al.^51^ presented a comprehensive data base for the entire country, however, one major limitation to their study was that the methods of collection were not uniform as this may have its effect on predictive data especially in areas where samples were not collected. Second, the data takes into account a 100-year data from published manuscripts. As it is, the vector dynamics is changing and the current events may have overtaken this, over a decade manuscript. To the best our knowledge, our study is the first to report model-based estimates of dominant malaria vectors for the whole of Nigeria, using contemporary data.

Members of *Anopheles gambiae* complex constituted the majority of the populations of *Anopheline*s collected during our study (occurring in all the 12 states) and may be responsible for malaria transmission within the country. This is not surprising as members of the complex have been implicated as the most important vectors of malaria in sub-Saharan. The complex has four species that occur in West Africa: *An. coluzzii, An. gambiae, An. arabiensis* and *An. melas*^52–54^.

*An. coluzzii, An. gambiae, An. arabiensis* were the members of the complex encountered in this study*.* This finding was somewhat similar to that of Okorie et al.^51^ who reported that *An. gambiae* complex, accounted for more than 60% of the overall composition of mosquitoes collected in their review. Some studies also buttressed the fact that the molecular S form (now referred to as *An. gambiae*) was predominant and had a wide distribution across Nigeria^55,56^. However, contrary to their findings, our work highlights the possibility of on-going species replacement from *An. gambiae* to *An. coluzzii* in the country. This could be a consequence of the recent efforts to increase LLIN distribution and use as well as possible changes in climatic conditions. These findings highlight the necessity for ongoing vector surveillance to provide data-based insight to control programmers and policy makers. Vector control strategies may need reshaping to target both of these major species. *An. coluzzii* has been reported to be an excellent vector for *Plasmodium malariae,* while both *An. coluzzii* and *An. gambiae* transmits both *P. falciparium* and *P. ovale* effectively^57^. Moreso, a study conducted in Benin suggested that *An. coluzzii* was more involved in malaria transmission in comparison with *An. gambiae,* which was previously believed to be the more efficient malaria vector^58^.

The importance of active vector surveillance cannot be overemphasized as it helps to understand how vector population thrives or changes over time, including the detection of new or invasive species. In this study, we also identified an invasive malaria vector; *An. stephensi* in one of the states in the northern part of the country^50^. *An. stephensi* is an excellent urban malaria vector and its presence in Nigeria is alarming owing to the behaviour of the species. Malaria in Africa has been essentially defined as a rural disease, but the establishment of *An. stephensi* population could result in a surge in cases of urban malaria^59,60^, putting at least 126 million people at risk^61^. Efforts to control malaria transmission in Nigeria have largely been concentrated on the members of the *An. gambiae complex* through deployment of LLINs due to the high predictability of their feeding behaviour (mostly anthropophilic) and ecology (strict breeding site preferences). It could be catastrophic if populations of *An. stephensi* have already established in the country, especially with the currently used control strategies in the country being targeted indoors.

Our model result suggests that the three sibling species; *An. coluzzii, An. gambiae* and *An. arabiensis* are widespread across all the ecological zones (from high to moderate), in a sympatric relationship, though the distribution of each of the species varied slightly. Of all the species, *An. arabiensis* seems to have the most restricted distribution, confined to two states in southern Nigeria and some portion of the northern parts of Nigeria.

The distribution of any disease vector including mosquito species are functions of the relationship that exists between factors such as climate, landforms, soil types and the human settlement patterns of different ecological zones^61–63^. However, this present study focused only on how climatic and topographic factors could affect the distribution pattern of major malaria vector in Nigeria. Mean temperature of the driest quarter, precipitation of the coldest quarter and annual mean temperature were strongly associated with the distribution of *An. arabiensis*. The ROC curve showed that warmer climates supported the breeding of *An. arabiensis* and are likely to be abundant in areas with lowest precipitation during the cooler part of the year. Other studies also suggested that *An. arabiensis* prefers warmer climates and thus limits their presence in cold swamps within the humid forest and high suitability in Sahel savanna localities^64–66^.

The high suitability observed for *An. gambiae* and *An. coluzzii* (more than two-thirds of the entire country is either highly or moderately suitable for both species) is also in keeping with the study of Akpan et al*.*^66^. It is important to note that areas with high suitability and low distribution density of both *An. gambiae* and *An. coluzzii* may have a serious likelihood of experiencing widespread prevalence alongside high distribution density, species migration and invasion^67^. Previous reports suggest that the range, relative abundance and ecological adaptability of *An. gambiae* complex are significantly influenced by seasonality, random temporal fluctuations and annual precipitation^62,65,68,69^. Therefore, their assertion agreed with our findings as all the aforementioned factors played a very crucial role in determining the distribution of the species in the study area. Our model suggests that higher temperatures and low precipitation influenced the presence of *An. coluzzii,* while the first three strongest environmental predictors for *An. gambiae* were related to temperature. The findings from our study indicate that certain environmental variables serve as common predictors for all three sibling species, while *An. gambiae* exhibits distinct environmental predictors not shared by the other species. This suggests that these variables play a unique role in influencing the distribution pattern of *An. gambiae* compared to the other mosquito species studied.

These findings highlight the importance of considering environmental factors, particularly climate and topography, in designing and implementing malaria control strategies in Nigeria. Understanding the distribution patterns and environmental preferences of mosquito species can help target when and where to deploy interventions, such as insecticide-treated bed nets, indoor residual spraying, and larval source management, especially to the area’s most at risk. For instance, larviciding in areas where precipitation is lower during the coolest part of the year may be highly impactful in controlling *An. arabiensis.* Additionally, monitoring and adapting control measures in response to changes in environmental conditions and vector behavior can enhance the effectiveness of malaria control efforts in the country.

One significant limitation of this study is the exclusion of parameters such as pH, dissolved oxygen, total dissolved solids, electrical conductivity, turbidity of the breeding sites, and demographic variables like the presence of cattle and human population density from our model. These variables are also crucial and may contribute to the explanation of mosquito species distribution. Further research should be conducted to incorporate these variables and evaluate their effects on the distribution of *Anopheles* mosquitoes in the country.

This study has, for the first time, predicted the potential distribution of the members of *Anopheles gambiae* complex across Nigeria using contemporary data involving standardized and uniform methods of mosquito collection. The Maximum entropy (MaxEnt) modeling used in this study is a general-purpose method for making predictions of inferences from incomplete information^33^. The model used in this study is a presence only modeling algorithm (i.e., absence data are not required). More so, the performance has been reported to be relatively better than other modeling methods^27,34^. The report that the model has been hardly influenced by small sample sizes with relatively robust prediction hence putting it among the top performing modeling tools^27^ made it the choice model for our study.

## Conclusion

Our study provides comprehensive data on malaria vectors in Nigeria. *An*opheles *gambiae* complex is the dominant vector, with species displacement observed. Active surveillance detects an invasive vector, *An*. *stephensi*, posing urban malaria risks. Varying environmental factors influence the distribution of different sibling species of the *An. gambiae complex*, requiring tailored control strategies. Active surveillance is very important in vector control. We have generated a model-based baseline species distribution of the major malaria vector population in the country using empirical data. Knowing that a country-wide mapping of vector distribution can be time consuming and expensive, hence the maps presented here could be used by the National Malaria Elimination Program to direct resources for vector control.

### Supplementary Information

Supplementary Table 1.

## Data Availability

The data sets in this study are available from the corresponding author on reasonable request.

## Abbreviations

MaxEnt: Maximum entropy
LLIN: Long lasting insecticidal net
IRS: Indoor residual spraying
LSM: Larval source management
WHO: World health organization
NMEP: National malaria elimination program
ENM: Ecological niche modeling
FCT: Federal capital territory
CDC: Center for diseases control
PCR: Polymerize chain reaction
LGA: Local government area
ROC: Receiver operating characteristics
AUC: Area under curve
PC: Percentage contribution
PI: Permutation importance
SRTM: Shuttle radar topography mission
GIS: Geographic information system
GPS: Global positioning system
